# Editorial: Emerging Concepts in Dengue Pathogenesis and Host Innate Immune Response

**DOI:** 10.3389/fcimb.2021.708484

**Published:** 2021-06-25

**Authors:** Binod Kumar, Yean K. Yong, Vikas Sood

**Affiliations:** ^1^ Department of Microbiology and Immunology, Loyola University Chicago, Chicago, IL, United States; ^2^ Laboratory Center, Xiamen University Malaysia, Malaysia, Sepang, Malaysia; ^3^ Department of Biochemistry, School of Chemical and Life Sciences, Jamia Hamdard, New Delhi, India

**Keywords:** Dengue virus, innate immunity, antiviral strategies, viral pathogenesis, inflammasomes

Dengue is one of the most rapidly advancing arthropod-borne viral disease present in over 100 countries. Dengue virus (DENV) infections are a serious health concern around the world. In a recent modelling‐based report it was estimated that around half of the world’s population is at the risk of this disease. Dengue fever is generally self‐limiting, however in certain cases it can progress to more complicated forms like Dengue Hemorrhagic Fever (DHF) and Dengue Shock Syndrome (DSS) with high mortality rates, as well as significant economic burdens. Moreover, subsequent infections of Dengue are more severe due to a phenomenon known as Antibody Dependent Enhancement (ADE) where pre-existing antibodies help the virus with different serotypes to infect monocytes more efficiently thereby contributing to the enhanced pathogenesis and spread of the disease.

Dengue viruses have evolved multiple strategies to escape the host innate immune system by both modifying viral RNA and viral protein or by post-translational modification of the host cellular proteins. The virus has been reported to trigger the host RLR as well as cGAS-STING axis to modulate the innate immune signaling in infected cells.

There are no specific therapies against this virus and thus novel antivirals to inhibit Dengue infections are highly desired. Although multiple strategies have been used to design vaccine candidates against this virus, these approaches have failed due to the complexity and epidemiology of the disease. To this end, there is an urgent need to identify novel cellular pathways and antiviral therapies that can be used to inhibit this virus.

The topics covered in this Research Topic helped gain insights into some of the very important aspects of Dengue biology. Hsieh et al. studied the effect of ageing on Dengue viral replication. They observed increased viral replication in human monocytic cells that were treated with D-galactose an inducer of cellular senescence. Further investigations revealed that induction of cellular senescence led to increased expression of DC-SIGN. Being one of the receptors for Dengue replication, increased expression of DC-SIGN following D-galactose treatment might contribute towards the enhanced replication of Dengue virus.

Another study by Zheng et al. tried to investigate molecular mechanisms contributing towards severe Dengue. Authors performed high throughput analysis on Dengue infected cells and identified several differentially regulated long non-coding RNAs. They focused on ERG-associated non-coding RNA (ERGAL lncRNA) as its expression was dependent on time post Dengue infection rather than viral dose. siRNA mediated knockdown of ERGAL suggested that it might regulate vascular endothelial barrier function *via* ERG. Dual luciferase data further suggested that ERGAL could bind to miR-183-5p thereby acting as a sponge and suppressing the effect of miR-183-5p. The study identified interplay among ERGAL and miR-183-5p and their effect in regulating vascular endothelial barrier functions.

The role of platelet cytokines in Dengue biology was reviewed by Singh et al. Authors provided an in-depth analysis of platelet biology and their fate during Dengue infection. They discussed platelet cell surface receptors contributing towards Dengue infection. Authors further discussed the role of various chemokines and cytokines including CCR1-CCL2 axis, CCR1-CCL5 axis, IL6, CXCL8, CXCL10, CXCL11 and IL1b following Dengue infection of platelets.

Apart from vascular leakage, neurological disorders are also one of the hallmark of Dengue virus infection. This aspect was studied by Shen et al. by infecting immunocompetent outbred ICR mouse model with Dengue virus. First of all the authors investigated the presence of Dengue virus following infection of ICR suckling mice. Among various organs, authors could successfully detect NS1 in liver and brain of infected mice though infection was very severe in brain as compared to liver. Then authors conducted single cell immune profiling of brain cells following Dengue infection and observed increased expression of NK-1 NK cells and levels of several cytokines including IL2, IL15, IL4, IL5, IL10, IL13IL1b, IL6 were increased. The data was in conjunction to human patients where transcriptomic studies revealed negative correlation of NK cells with viral load. The study provides insights into the possible mechanism of immune erosion in Dengue patients.

Antibody dependent enhancement contributes towards Dengue severity in pre-exposed individuals. The phenomenon was extensively reviewed in two different reports by Shukla et al. and Narayan and Tripathi. Shukla et al. discussed the biology of DENV, the antibody response against DENV, and the challenges of vaccine development against DENV. In addition, they have proposed have EDIII as a dengue vaccine candidate that is capable of eliciting higher type-specific antibodies with lower ADE potential. Similarly, the article by Narayan et al. outlines the main concept of intrinsic ADE during DENV infection that contributes to the severe dengue pathogenicity. The article provides a good overview on the literature of *in vitro* and cell-signaling studies and propose that drugs against effectors of intrinsic ADE could be used as prophylactic treatment options to minimize disease severity in both adults and infants infected with DENV.

Recent advances on DENV research have shown the role of inflammasomes in contributing towards disease severity. Here, Shrivastava et al. discussed the contribution of inflammasomes in fueling Dengue severity. Authors discussed activation and induction of NLRP3 (one of the widely studied inflammasome) following viral infections. Moreover, inflammasomes specific to various cells including macrophages, dendritic cells, and platelets were also discussed. Apart from this, Dengue ADE and Dengue protein mediated inflammasome induction was also extensively discussed.

Dengue viral proteins have been shown to interact with various cellular proteins. Virus host interactions of one of the most important Dengue protein NS5 was discussed by Bhatnagar et al. They compiled various studies involving Dengue NS5 and host protein interactions to understand the interactome of the viral protein. Their analysis revealed that Dengue-NS5 associated with several host proteins that were involved in various cell signaling pathways including JAK-STAT, RNA processing, cell-cycle, protein synthesis and processing. They concluded that Dengue NS5 interacting partners could be exploited to design antiviral drugs against DENV.

Innate immune responses have been shown to be critical in regulating viral infections. However, viruses have been shown to deploy various strategies to evade host innate immune responses. Innate immune responses against Dengue virus were discussed by King et al., in their review article, have discussed extensively about the inflammatory responses to DENV infection due to innate immune cells, their effector functions and clinical course of the disease. They have talked about the DENV infection in two waves; in the first wave the tissue-resident phagocytes and keratinocytes are productively infected; while in the second wave the monocyte-derived macrophages and DCs are recruited and infected. They have also discussed how the DENV have evolved to both escape from the pre-existing adaptive immunity from antibodies as well as from host innate immune responses.

Several innate immune cells play critical role in regulating Dengue infections. Here Malavige et al. studied the fate of various innate immune cells following Dengue infections. Monocyte transcriptomics from Dengue patients revealed the importance of these cells in shaping Dengue severity. Monocytes are also involved in Dengue ADE which leads to suppression of interferon system in these cells. Secretion of various mediators by mast cells following Dengue infection has been implicated in vascular leakage which was also discussed in this article. The fate of T cells in Dengue severity was also debated in the article.

Lastly, Non-coding RNAs have been recently implicated in regulating host responses. Rajput et al. discussed the role on non-coding RNAs in regulating Dengue pathogenesis. These non-coding RNAs could either directly modulate the virus by binding to its genome or indirectly regulate viral pathogenesis by modulating host innate immune responses. The role of non-coding RNAs in Dengue severity was also discussed by the authors.

Overall, this Research Topic shed light on the current research advancements made in the area of dengue virus research. While it is imperative to have an in-depth understanding of the host innate immune response to dengue virus infection, it is more important to understand how viruses have evolved to evade these responses and establish successful infection. A better understanding of the host pathogen interactions is fundamental to the discovery and implementation of antiviral strategies, the development of candidate vaccines against dengue viruses.

## Author Contributions

All authors contributed to the article and approved the submitted version.

## Conflict of Interest

The authors declare that the research was conducted in the absence of any commercial or financial relationships that could be construed as a potential conflict of interest.

